# Digital twins in sports science: applications for performance enhancement, injury prevention, and rehabilitation through advanced big data analytics and deep learning methodologies – a comprehensive narrative review

**DOI:** 10.5114/biolsport.2026.161106

**Published:** 2026-04-20

**Authors:** Afef Sédiri, Sabri Barbaria, Halil Ibrahim Ceylan, Andrea de Giorgio, Luca Puce, Valentina Stefanica, Nicola Luigi Bragazzi, Ismail Dergaa, Hanene Boussi Rahmouni

**Affiliations:** 1 Laboratory of Biophysics and Medical Technologies, LR13ES07 (BTM), University of Tunis Elmanar, Higher Institute of Medical Technologies of Tunis (ISTMT), Tunis, Tunisia; 2 Physical Education and Sports Teaching Department, Faculty of Sports Sciences, Atatürk University, Erzurum 25240, Turkey; 3 Artificial Engineering, Naples 80121, Italy; 4 Department of Neuroscience, Rehabilitation, Ophthalmology, Genetics, Maternal and Child Health, University of Genoa, 16132 Genoa, Italy; 5 Faculty of Sciences, Physical Education and Informatics, National University of Science and Technology Politehnica Bucharest, Pitesti University Center, Pitesti, Romania; 6 Laboratory for Industrial and Applied Mathematics (LIAM), Department of Mathematics and Statistics, York University, Toronto, ON M3J 1P3, Canada; 7 High Institute of Sport and Physical Education of Ksar Said, University of Manouba, Mannouba 2010, Tunisia

**Keywords:** Artificial intelligence, Big data analytics, Biomechanics, Convolutional neural networks, Deep learning, Digital technology, Injury prevention, Machine learning, Rehabilitation, Sports medicine, Virtual reality

## Abstract

Digital twin (DT) technology, combined with advanced computational methodologies, represents a paradigm shift in sports science. DTs generate virtual athlete replicas through real-time data integration and predictive analytics. While computational capabilities and data acquisition have advanced rapidly, DT applications in sports remain fragmented, warranting systematic synthesis. This narrative review examines the applications of DT in sports, with a focus on big data analytics and deep learning for performance enhancement, injury prevention, and rehabilitation. A narrative review was conducted using publications from 2018 to 2025 across multiple databases. Eligible studies applied DT frameworks, Artificial Intelligence (AI), including machine learning algorithms and deep neural networks, or big data analytics in elite and amateur sport contexts, with a focus on football as a case study. Extracted data focused on technological approaches, clinical outcomes, and practical applications. DT applications cluster into three domains: (1) performance enhancement via biomechanical modelling (convolutional or recurrent neural networks); (2) injury prevention using ensemble learning and predictive risk models; and (3) rehabilitation optimization through multimodal sensors and virtual reality. Key examples include cycling telemetry, computer vision for technique correction, and real-time musculoskeletal monitoring. The integration of generative AI and the Internet of Things further enhances predictive accuracy and decision-making. DTs offer significant potential for proactive athlete management. Widespread adoption requires standardized protocols, clinical validation, and robust ethical frameworks for data privacy. Successful integration supports data-driven training, individualized recovery, and enhanced athlete welfare.

## Graphical abstract

**FIG:**
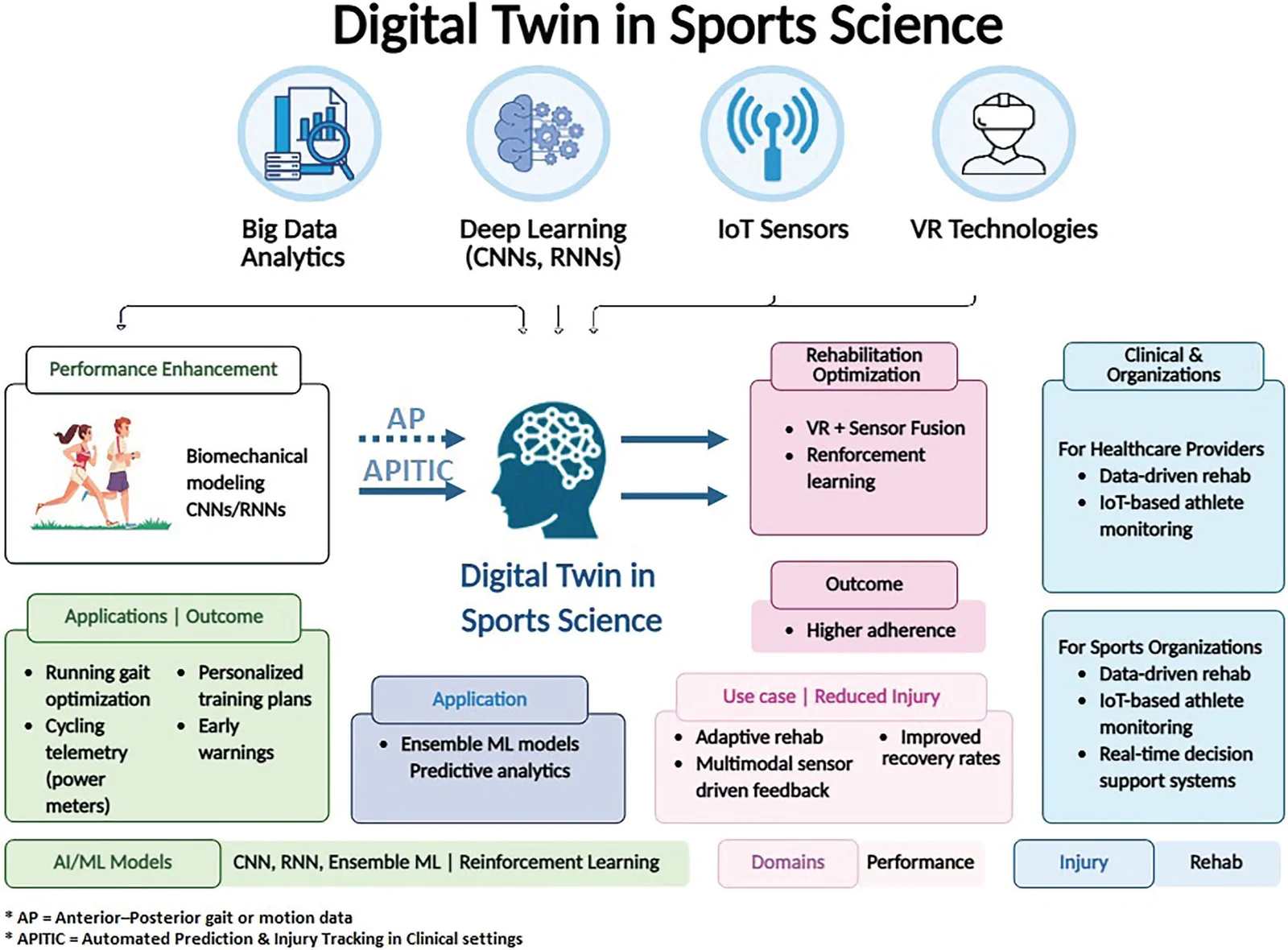
Graphical Abstract

## INTRODUCTION

The contemporary sports industry generates annual revenue exceeding $417 billion globally and has recently undergone a technological transformation driven by the convergence of advanced computational methodologies and sophisticated data acquisition systems [1]. This economic magnitude highlights the critical importance of marginal performance gains.

Digital twin (DT) technology provides an innovative alternative to traditional coaching methods, shifting toward evidence-based, data-driven optimization strategies [2]. The epidemiological burden of sports-related injuries affects approximately 3.5 million pediatric and adolescent athletes annually in the United States, with associated direct medical expenditures exceeding $1.8 billion, thereby necessitating cutting-edge preventive approaches [3]. The economic ramifications extend well beyond immediate healthcare costs and include prolonged rehabilitation expenses, productivity losses, and substantial reductions in quality-of-life indices [4].

Elite athletic performance in contemporary competitive environments increasingly requires detailed monitoring and optimization methodologies, as marginal improvements often determine the difference between success and failure [5]. Traditional coaching paradigms retain considerable value through experiential knowledge; however, they face inherent limitations in delivering real-time, personalized feedback and predictive analytics [6]. The technological evolution of sports has progressed from isolated performance-tracking systems to integrated athlete-monitoring ecosystems that incorporate Internet of Things (IoT) sensor networks, edge computing platforms, and cloud-based analytics infrastructures [7, 8].

DT technology, originally developed for industrial manufacturing applications, has emerged as a tool for personalized monitoring in sports science through the creation of dynamic virtual replicas that continuously mirror real-time athletic performance and physiological responses [9]. It is essential to distinguish between Artificial Intelligence (AI)-enabled sports analytics and DT systems. While the former typically involves one-way data processing for task-specific analyses, DT requires continuous, bidirectional synchronization in which the virtual model evolves alongside the athlete’s physiological and biomechanical state in real time. This advancement directly addresses key gaps in modern sports medicine, including personalized training optimization, injury-risk prediction, and evidence-based rehabilitation protocols [1, 10].

Contemporary DT frameworks rely on advanced machine-learning architectures, including convolutional neural networks (CNNs) for biomechanical pattern recognition, recurrent neural networks (RNNs) for temporal analyses, and ensemble methods for injury-risk prediction [2, 11]. This computational sophistication enables realtime monitoring of multiple physiological parameters, such as heart rate variability, electromyographic muscle activation, three-dimensional joint kinematics, and metabolic response indicators [5, 12]. Integration with virtual reality (VR) further enhances DT capabilities by enabling immersive training environments and rehabilitation protocols, thereby fostering a synergistic relationship between physical and virtual training modalities [8, 13]. The convergence of AI and IoT technologies in sports science thus offers substantial opportunities for personalized athletic development and evidence-based injury-prevention strategies [9, 14].

Despite these advances, current research reveals significant implementation gaps across sports disciplines. There is limited standardization of data collection protocols and validation methodologies [1, 15]. Moreover, the absence of comprehensive longitudinal studies assessing long-term effectiveness and clinical outcomes represents a critical knowledge gap that warrants systematic investigation [16]. Inadequate evaluation of cost-effectiveness and accessibility barriers further constrains widespread adoption across diverse organizational contexts [3, 17]. Limited attention to data privacy and security in athletic settings raises important ethical concerns [18]. Finally, the lack of standardized metrics for evaluating DT effectiveness across sports hampers comparative analyses [6, 19], while insufficient examination of user acceptance and adoption barriers among athletes and coaching staff poses additional implementation challenges [7, 20].

This comprehensive narrative review aimed to examine current DT applications in sports, assessing their effectiveness in enhancing performance and preventing injuries through advanced computational approaches, and to identify priorities for future research to support evidence-based implementation. Studies demonstrating bidirectional data flow between the physical athlete and the digital model were prioritized, whereas generic AI applications lacking a feedback loop were excluded to maintain focus on the core DT paradigm.

## METHODOLOGY AND BIG DATA ANALYTICS FRAMEWORK

### Comprehensive Literature Search Strategy

This narrative review followed established guidelines for narrative synthesis and incorporated structured elements to ensure comprehensive coverage and methodological rigor. Multiple electronic databases were searched, including PubMed/MEDLINE, Scopus, Web of Science Core Collection, IEEE Xplore Digital Library, ACM Digital Library, and SportDiscus with Full Text. Grey literature was identified through Google Scholar and preprint servers, including arXiv and bioRxiv.

The search strategy was developed in consultation with a medical librarian and piloted across databases, using combinations of controlled vocabulary terms (MeSH terms, where applicable) and free-text keywords.

Primary search terms included “digital twin*” OR “digital replica*” OR “virtual twin*” OR “cyber-physical system*,” combined with “sport*” OR “athletic*” OR “biomechanic*” OR “exercise*” OR “physical activity,” and further refined using “machine learning” OR “deep learning” OR “artificial intelligence” OR “neural network*” OR “predictive analytic*.” Secondary search terms focused on “performance enhancement” OR “injury prevention” OR “rehabilitation” OR “recovery,” combined with “big data” OR “data analytics” OR “IoT” OR “wearable*” OR “sensor*.” Inclusion criteria encompassed peer-reviewed publications in English published between January 2018 and December 2025; studies involving DT applications in sports contexts; research examining performance enhancement, injury prevention, or rehabilitation; studies employing machine learning, AI, or big data analytics; and both original research and review articles. Exclusion criteria comprised non-English publications, conference abstracts without full-text availability, case reports with fewer than five participants, studies focusing exclusively on non-human subjects, and publications prior to 2018 to ensure a contemporary technology focus [21, 22].

### Big Data Analytics and Computational Framework

The theoretical and analytical framework for this narrative review was grounded in the principles of big data analytics, acknowledging that contemporary sports generate massive volumes of heterogeneous data that require sophisticated computational approaches [12, 23]. The review was structured around the established “5 V’s” paradigm, categorizing data sources according to “Volume” (terabytes of sensor data generated daily), “Velocity” (real-time streaming and processing requirements), “Variety” (multimodal sensor types), “Veracity” (data quality and reliability), and “Value” (generation of actionable insights) [13, 24] (Table 1).

**TABLE 1:** Characteristics of Big Data in Sports Digital Twin Applications according to the 5V framework

Dimension	Sports Context	Technical Specifications	Computational Requirements
Volume [25]	50GB-2TB per athlete/day	Multi-sensor data streams	Distributed storage systems
Velocity [26]	1000Hz+ sampling rates	Real-time processing	Edge computing capabilities
Variety [25]	IMU, GPS, video, and physiological	Heterogeneous data formats	Data fusion algorithms
Veracity [27]	Sensor accuracy ± 2–5%	Quality assurance protocols	Outlier detection methods
Value [9]	Performance optimization	Predictive model accuracy	Machine learning pipelines

Abbreviations: IMU: Inertial Measurement Unit; GPS: Global Positioning System; Hz: Hertz; GB: Gigabytes; TB: Terabytes.

### Deep Learning Architecture Analysis

Our analytical approach entailed a comprehensive examination of the deep learning architectures employed in sports-related DT applications, with particular emphasis on CNNs for spatial pattern recognition and RNNs for temporal sequence modeling [22, 28]. We systematically analyzed implementation characteristics, including network architectures, training strategies, loss functions, optimization algorithms, and performance metrics across diverse sporting contexts [11, 29]. This review further examined three principal deep learning paradigms: supervised learning for labeled classification of sports movements, unsupervised learning for pattern discovery in athlete behavior, and reinforcement learning for the development of optimal strategies [14, 30]. Each paradigm was evaluated with respect to computational complexity, predictive accuracy, generalization performance, and practical feasibility of implementation in real-world sports settings [21, 31].

### Data Synthesis and Quality Assessment

Two independent reviewers conducted the initial screening of titles and abstracts, and full-text articles were retrieved for studies deemed potentially eligible. Any disagreements were resolved through discussion with a third reviewer when necessary. A structured flow diagram was maintained to document the study selection process in accordance with established narrative review protocols. Standardized data extraction forms were developed, piloted, and applied to collect information on study characteristics (design, sample size, and population) and on technological specifications (DT architectures and algorithms), intervention details, outcome measures, and key findings. Data extraction was performed by one reviewer and independently verified by a second reviewer to ensure accuracy and completeness.

Given the heterogeneity of study designs and the narrative nature of the review, a modified quality assessment framework was applied that integrated elements of technology assessment, methodological evaluation, reporting quality, and clinical relevance. Methodological rigor was assessed based on the clarity of objectives, appropriateness of the study design, and adequacy of the sample size. Technical validation was assessed based on reported algorithm performance metrics, the use of validation datasets, and comparisons with established reference standards. Reporting completeness was assessed based on the level of detail provided to ensure reproducibility and adherence to statistical reporting standards, while clinical applicability was determined by practical relevance, safety considerations, and the feasibility of real-world implementation. Potential sources of bias, including selection, measurement, and reporting bias, were also systematically considered. A narrative synthesis approach was adopted due to substantial heterogeneity in study designs, technological implementations, and outcome measures. Findings were organized thematically according to major application domains, including performance enhancement through advanced analytics, injury prevention *via* predictive modeling, and rehabilitation optimization through intelligent systems [16, 32].

A convergent synthesis design was employed, integrating quantitative evidence, such as algorithm performance metrics and clinical outcomes, with qualitative findings on user experience and implementation barriers to provide a comprehensive and balanced interpretation. Confidence in the synthesized evidence was assessed using principles adapted from established quality assessment frameworks, taking into account methodological limitations, coherence across studies, data adequacy, and relevance to the review objectives.

While the synthesis and quality assessment provided a structured overview of the included studies, it is important to acknowledge the methodological shortcomings inherent to a narrative review.

### Limitations of the Narrative Review

As previously mentioned, this review employed a narrative synthesis methodology, which enabled a broad, integrative discussion of emerging technologies in sports DT and big data analytics. While this approach supported comprehensive exploration of conceptual frameworks, methodological diversity, and applied use cases, it did not provide the statistical rigor, formal bias assessment, or reproducible search methodology inherent to systematic reviews [21, 22]. Consequently, conclusions drawn here are indicative rather than definitive, highlighting trends, gaps, and potential applications rather than providing quantifiable effect sizes or evidence hierarchies. Research questions requiring precise effect estimation, meta-analysis, or rigorous comparative evaluation across studies would necessitate systematic review methodologies with pre-specified inclusion/exclusion criteria, quality assessment tools, and formal synthesis protocols.

## DEEP LEARNING AND CONVOLUTIONAL NEURAL NETWORKS IN SPORTS PERFORMANCE ENHANCEMENT

### Advanced CNN Architectures for Biomechanical Analysis

Modern sports performance analysis has been substantially advanced by sophisticated CNN architectures that process complex spatiotemporal biomechanical patterns with high accuracy [28, 33]. Current implementations, particularly three-dimensional CNNs (3D-CNNs), have demonstrated strong performance in analyzing basketball technical actions, with reported classification accuracies ranging from Network: SE-ResNet. 93% to 98%, depending on the architecture, dataset size, and recording conditions (Table 2). For example, 3D-CNNs achieved 93.1% accuracy on NBA SportVU video sequences recorded at 30 frames per second [33], while SE-ResNet-CNNs reached 98% in general sports classification tasks [37]. However, these performance levels largely reflect performance on controlled or curated datasets, and reliability often declines when applied to smaller, custom-built datasets or to unconstrained field environments characterized by noise, occlusion, and variability in recording conditions [29, 34]. The architectural progression from conventional two-dimensional CNNs to advanced 3D-CNN frameworks enables the simultaneous extraction of spatial and temporal features inherent to athletic movements [30, 35]. Beyond increased architectural complexity, the primary value of 3D-CNNs in sports science lies in their capacity to automate the identification of joint coordination patterns and movement efficiency metrics, thereby transforming raw video data into actionable biomechanical signatures for performance optimization and injury risk assessment [33, 36].

**TABLE 2:** Performance and technical specifications of CNN-based architectures for sports motion analysis.

Architecture	Sport Application	Accuracy (%)	Processing Speed (FPS)	Key Features
3D-CNN + LSTM [33]	Basketball analysis	93.1	30	Spatiotemporal modelling
SE-ResNet-CNN [37]	General sports classification	98	45	Attention mechanisms
Multi-scale CNN [38]	Swimming technique	94.02	25	Multi-resolution analysis
Hybrid LSTM-CNN [39]	Gait analysis	97.11	20	Temporal sequence learning

Abbreviations: FPS (Frames Per Second) values are benchmarks based on the cited hardware and may vary significantly with software optimizations like TensorRT; CNN: Convolutional Neural Network; LSTM: Long Short-Term Memory; Squeeze-and-Excitation Residual Network: SE-ResNet.

The integration of attention mechanisms, particularly squeeze-andexcitation (SE) networks, substantially enhances CNN performance by dynamically recalibrating feature map responses according to their relative informational importance [34, 40]. This architectural refinement enables neural networks to allocate computational resources preferentially to biomechanically salient features while attenuating irrelevant background signals, thereby improving classification accuracy and reducing computational overhead [35, 41].

### Real-time Performance Monitoring Through Big Data Analytics

DT technology leverages big data analytics to integrate multimodal sensor streams, providing a synchronized and holistic representation of athlete status that surpasses traditional isolated monitoring approaches [23, 42]. Contemporary implementations combine multiple sensor modalities, including inertial measurement units (IMUs) sampled at frequencies exceeding 1000 Hz, global positioning systems (GPS) with centimetre-level accuracy, and high-definition video captured at up to 240 frames per second. The data-processing pipeline relies on advanced edge-computing architectures to perform initial signal filtering and noise reduction locally, thereby minimizing latency and enabling near–real-time feedback [42, 43]. Professional cycling represents a mature example of real-time DT deployment. In this context, DT systems process power-output measurements with an accuracy of ± 1% across typical competitive ranges of 200–1000 W [44, 45]. By integrating environmental variables, such as wind velocity and barometric pressure, with physiological stress indicators, these frameworks support predictive pacing strategies and enable forecasting of performance degradation under varying race conditions [46, 47].

**FIG. 1:**
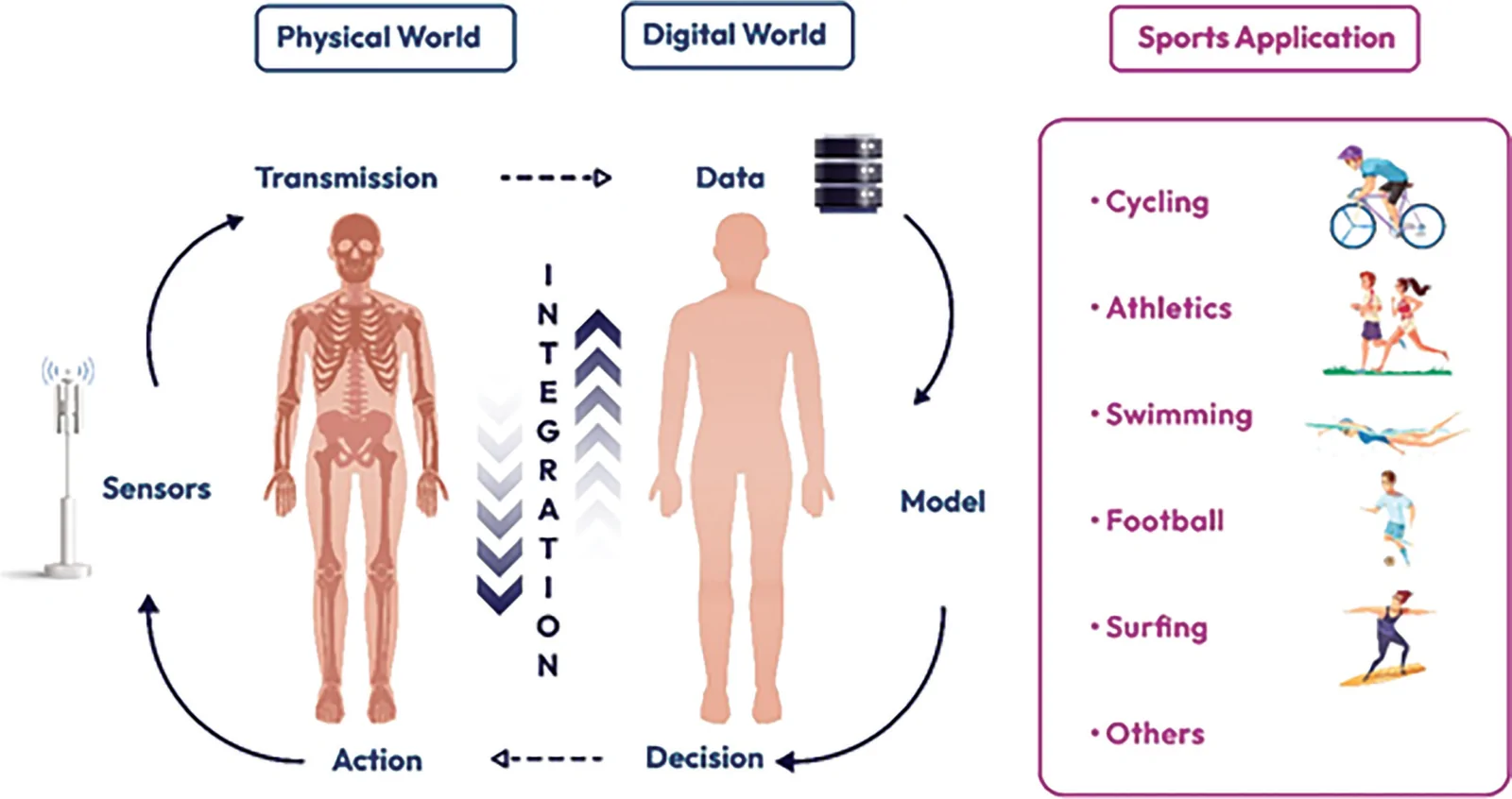
Integration of the Physical and Digital Realms through a Digital Twin Framework in Sports Science.

**FIG. 2:**
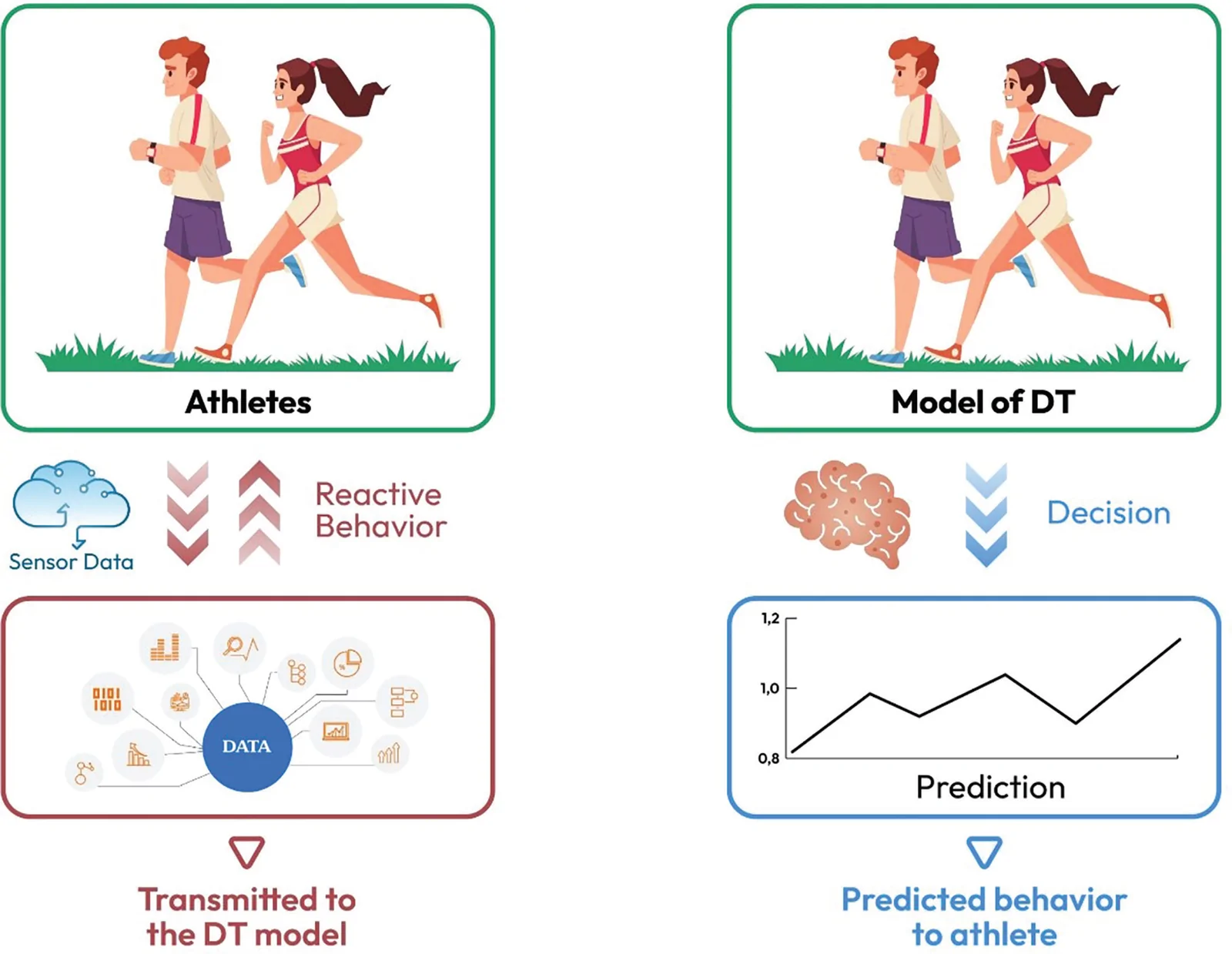
Reciprocal Feedback Mechanism between Athlete and Digital Twin for Predictive Decision-Making. Abbreviation: DT: Digital Twin.

As depicted in Figure 1, the DT framework in sports science establishes a continuous, closed-loop interaction between the physical athlete and the corresponding virtual model through sensor-driven data acquisition, computational modeling, and real-time decision support. The process begins with the capture of biomechanical and physiological signals in the physical domain, which are transmitted to the digital domain for integration, processing, and dynamic modeling of athlete behavior and performance. Insights generated by the digital model are then translated into data-driven recommendations that are communicated back to the athlete, guiding physical actions and optimizing performance outcomes. This bidirectional feedback loop enables personalized interventions, proactive injury prevention, and performance enhancement. Demonstrated applications span multiple sports, including cycling, athletics, swimming, football, and surfing, highlighting the broad applicability of DT systems across diverse athletic disciplines. Collectively, this integration represents a cyber-physical system in which digital intelligence and physical performance co-evolve, supporting optimized training processes and improved sports medicine outcomes (Figure 2).

### Computer Vision and Deep Learning for Technique Optimization

Advanced computer vision algorithms powered by deep CNNs enable detailed biomechanical analyses that substantially surpass traditional video-based movement assessment methodologies [36, 48]. The integration of three-dimensional motion-capture data with forceplatform measurements and high-density electromyographic recordings yields comprehensive movement profiles suitable for fine-grained technique optimization [40, 49]. Using automated pose estimation, the computational pipeline identifies anatomical landmarks and tracks subtle deviations in technique.

When analyzed using RNNs, these deviations provide longitudinal insights into performance decrements that are often imperceptible to visual inspection alone [48, 49].Weightlifting applications have demonstrated particular effectiveness in technique optimization, with DT systems delivering precise feedback on barbell trajectory, joint angular velocities, and force application patterns throughout lift execution [50, 51].

Computer vision algorithms perform real-time movement analysis by benchmarking athletes’ performance against optimal-technique models derived from large-scale biomechanical databases that encompass thousands of elite athlete performances [52, 53]. The fusion of kinetic and kinematic data streams generates integrated performance profiles that inform progressive training adaptations through evidence-based recommendations [51, 54].

VR integration further enhances training by providing immersive feedback environments that accelerate motor learning and skill retention [13, 55]. These systems employ haptic feedback mechanisms synchronized with visual displays to create multisensory learning experiences that engage multiple neural pathways concurrently [55, 56].

### Personalized Training: Bridging Machine Learning and Coaching Practice

DT technology supports the development of highly individualized training protocols that adapt dynamically to athlete-specific responses through advanced machine learning algorithms [46, 57]. Within this computational framework, ensemble learning methods are employed to optimize training prescriptions; however, the principal value of DT systems resides in their capacity to translate complex analytical outputs into actionable decision support for practitioners.

In team sports such as football or rugby, DT platforms integrate real-time GPS and IMU data to continuously monitor external load profiles. Strength and conditioning specialists can leverage DT outputs to detect meaningful deviations from an athlete’s historical load baseline, enabling immediate adjustments to training volume and intensity to mitigate the risk of overreaching and injury. In endurance disciplines, including cycling and athletics, DT systems augmented with computational fluid dynamics simulations are used to evaluate aerodynamic efficiency. These models allow coaches to identify optimal racing postures that maximize speed while maintaining physiological comfort and sustainability under competitive conditions. At the physiological level, DT frameworks increasingly incorporate reinforcement learning approaches to iteratively refine recommendations based on real-time feedback [58, 59]. This adaptive process enables medical and performance staff to simultaneously monitor Twin. sleep architecture, nutritional intake, and autonomic nervous system responses, thereby supporting a more holistic and responsive approach to athlete recovery and readiness [42, 60].

By converting complex machine-learning outputs into precise, context-aware recommendations, DT systems enable a shift from reactive training paradigms to proactive, anticipatory performance management strategies.

## PREDICTIVE ANALYTICS AND MACHINE LEARNING FOR INJURY PREVENTION

### Advanced Machine Learning Models for Injury Risk Assessment

Machine learning algorithms enable proactive injury intervention by developing comprehensive risk prediction models [31, 61]. Contemporary implementations commonly rely on ensemble learning approaches, including random forests, gradient-boosting machines, and support vector machines, which have demonstrated strong predictive performance for specific injury outcomes across multiple sports contexts [61, 62] (Table 3).

**TABLE 3:** Predictive performance of machine learning models for sports injuries.

Algorithm Type	Injury Category	Sensitivity (%)	Specificity (%)	AUC Score	Methodological Context / Limitations
SVM (binary classifier) [65]	General sports injury	92.5	96.0	Not reported	Real-time monitoring system; good classification metrics, but no AUC provided

Logistic/ML (decision tree) [66]	Lower-limb injuries, general sports injury	55.6	74.2	66.3	Youth football players are limited by lower AUC and imbalanced data

Interpretable SVM [67]	Football injury risk classification	Not reported	Not reported	99.2 (ROC-AUC)	Excellent AUC; potential dataset overfitting cautions noted

Abbreviations: AUC: Area Under the Curve; SVM: Support Vector Machine; ML: Machine Learning.

Model evaluation within these frameworks extends beyond overall accuracy and emphasizes sensitivity and the area under the receiver operating characteristic curve (AUC) to appropriately capture the low-incidence and imbalanced nature of injury events. These models process high-dimensional data streams that integrate biomechanical variables (e.g., joint kinematics and ground reaction forces), physiological parameters (e.g., heart rate variability and neuromuscular fatigue indicators), and contextual factors (e.g., training load history and environmental conditions) [62, 63]. Advanced feature engineering methods are applied to extract informative representations from raw sensor signals, while dimensionality reduction techniques enhance computational efficiency and model generalizability [63, 64].

Deep learning architectures, particularly LSTM networks, are wellsuited for modelling temporal dependencies underlying injury development patterns [52, 68]. These RNNs process sequential data over extended time horizons, enabling the detection of subtle progression signals that may precede injury onset by days or weeks [68, 69]. Their predictive scope extends beyond short-term injury risk estimation to encompass longer-term health trajectories and projections of career longevity [69, 70].

Beyond architectural performance, critical examination of the high accuracy rates reported in contemporary sports machine learning models reveals a substantial risk of data leakage [71]. In several reviewed studies, temporal dependencies or participant overlap between training and testing datasets are insufficiently controlled, a limitation compounded by the absence of standardized validation protocols [72, 73]. As a result, reported performance metrics often reflect laboratory-optimized conditions and fail to generalize to independent athletic cohorts, highlighting the persistent gap between theoretical modelling and real-world implementation [2, 74]. Moreover, the frequent reliance on small and homogeneous samples (often fewer than 20 participants) substantially limits statistical power and external validity [75], thereby constraining applicability to underrepresented populations, including female athletes and youth cohorts [8].

These methodological limitations have direct implications for the reliability of the reported performance metrics and the readiness of these models for real-world implementation [2, 74]. In particular, data leakage and insufficient separation of training and testing datasets may lead to systematic overestimation of predictive accuracy, giving a misleading impression of model robustness [71–73]. Similarly, the frequent use of small, homogeneous samples substantially reduces statistical power and external validity [75], thereby limiting generalizability to broader athletic populations, including female athletes and youth cohorts [8]. As a result, many models that demonstrate high accuracy or AUC in controlled laboratory settings may require substantial revalidation before deployment in applied sports medicine or performance monitoring contexts.

### Big Data Analytics for Load Management and Recovery Optimization

DT technology has transformed load management practices by enabling comprehensive monitoring and analysis of training stress and recovery status within big data analytics frameworks [23, 76]. These systems integrate external load metrics, including distance covered, sprint frequency, and acceleration profiles, with internal load indicators such as heart rate responses, perceived exertion scales, and biochemical markers, thereby generating holistic representations of athlete load exposure [76, 77]. Machine learning algorithms leverage these integrated profiles to identify optimal load–recovery ratios by analyzing individual response patterns alongside population-level databases comprising millions of recorded training sessions [77, 78]. Real-time load monitoring supports dynamic adjustment of training volume and intensity, preventing excessive fatigue accumulation while maximizing adaptive physiological responses [78, 79].

Predictive models further incorporate circadian rhythm influences, environmental conditions, and psychological stress indicators to optimize training timing and intensity on an individualized basis [77, 80]. Recovery optimization constitutes a central component of injury prevention strategies. Within DT frameworks, recovery status is monitored through multimodal sensing, including sleep architecture assessment *via* actigraphy and polysomnography, autonomic nervous system evaluation through heart rate variability analysis, and biochemical recovery markers obtained through noninvasive sensing technologies [79, 81]. These comprehensive monitoring approaches enable identification of individualized recovery patterns and optimal training windows using personalized algorithms that account for genetic predispositions, training history, and lifestyle-related factors [82, 83].

Despite these advances, the predictive performance of current injury risk models, summarized in Table 2, warrants cautious interpretation. Although several studies report high sensitivity and specificity, often exceeding 55% for anterior cruciate ligament injuries and muscle strains, such estimates are frequently derived from controlled experimental environments or rely on synthetic data balancing techniques to address the rarity of injury events. As DT technology continues to mature, future frameworks should prioritize precision–recall–based evaluation metrics and prospective validation in real-world athletic cohorts to ensure that reported predictive performance translates into effective clinical decision-making and meaningful injury mitigation in applied settings.

### Computer Vision and Movement Quality Assessment

DT technology enables advanced assessment of movement quality through computer vision algorithms that identify subtle biomechanical deviations associated with increased injury risk [48, 84]. Contemporary pose estimation methods process video data at high temporal resolution, extracting three-dimensional joint coordinates and computing kinematic variables with accuracy approaching that of laboratory-based motion capture systems [84, 85].

Within this computational pipeline, CNNs trained on extensive databases encompassing both normal and pathological movement patterns are employed to detect asymmetries, compensatory strategies, and movement dysfunctions [85, 86]. A defining advantage of the DT framework is its capacity for continuous longitudinal monitoring, enabling detection of progressive deterioration in movement quality that often precedes the onset of acute injury. This capability supports proactive intervention through real-time modification of techniques or targeted load adjustments [86, 87].

When integrated with DT technology, functional movement screening protocols are transformed from largely observational tools into objective, data-driven assessments based on quantifiable metrics [87, 88]. The fusion of kinematic analysis with force production data yields detailed movement profiles that inform personalized intervention strategies and enable longitudinal tracking of rehabilitation progress and functional recovery [89, 90].

### Quality, Reproducibility, and Validation Challenges

While the integration of DT technology in sports demonstrates substantial promise, several critical challenges persist regarding the strength and reliability of the current evidence base. As previously mentioned, a considerable proportion of the reviewed studies relies on small cohorts of elite athletes, often comprising fewer than 20 participants. Although such investigations provide high-fidelity, wellcontrolled data, their limited statistical power limits generalizability to broader athletic populations, including amateur and developmental levels.

In parallel, the widespread use of complex deep learning models, particularly within proprietary or commercial DT systems, raises concerns related to interpretability and reproducibility. The scarce availability of open-access datasets, coupled with insufficient reporting of model architectures and hyperparameters, poses a major barrier to independent replication of the high accuracy rates frequently reported in primary studies. Moreover, most validation efforts remain confined to controlled laboratory settings, whereas real-world sporting environments introduce substantial sources of variability, such as fluctuating lighting conditions for computer vision systems and sensor displacement due to sweat or movement. These factors often lead to significant performance degradation when DT models are deployed in the field, underscoring the gap between experimental validation and real-world deployment.

Additional challenges emerge in the context of injury prediction, where models targeting outcomes such as anterior cruciate ligament injuries exhibit high sensitivity in some studies but fail to achieve clinical relevance in others. These inconsistencies are frequently attributable to severe class imbalance inherent to injury data and the uncritical application of synthetic data balancing techniques, such as the Synthetic Minority Over-sampling Technique (SMOTE), which may artificially inflate performance estimates [71, 75]. As a result, reported accuracy metrics often fail to persist under longitudinal monitoring conditions. Future DT frameworks should therefore prioritize evaluation metrics based on precision–recall relationships rather than overall accuracy, alongside prospective validation in real-world environments, to more accurately reflect true predictive performance and clinical utility in applied sports settings [2, 74].

## DEEP LEARNING APPLICATIONS IN SPORTS REHABILITATION AND RECOVERY

### Virtual Reality and Immersive Rehabilitation Environments

The integration of DT technology with VR systems enables advanced immersive rehabilitation environments that enhance patient engagement and therapeutic outcomes through evidence-based protocols [55, 91]. These systems leverage real-time biomechanical feedback to generate interactive virtual environments that adapt dynamically to patient performance, thereby delivering personalized rehabilitation experiences optimized for motor learning and functional recovery [91, 92].

The underlying computational architecture relies on advanced rendering engines capable of producing photorealistic virtual environments at frame rates exceeding 90 frames per second, a requirement for minimizing motion sickness and sustaining immersion [92, 93].

Coupling VR platforms with motion capture systems allows precise tracking of patient movements with sub-millimeter accuracy, supporting detailed assessment of rehabilitation progress and finegrained technique refinement [93, 94].

Contemporary VR-based rehabilitation platforms further incorporate haptic feedback mechanisms synchronized with visual stimuli, creating multisensory learning experiences that promote neuroplasticity and accelerate functional recovery [56, 95]. In addition, the gamification features inherent to VR applications substantially improve patient motivation and adherence. As summarized in Table 4.

**TABLE 4:** Summary of VR-assisted rehabilitation outcomes across common sports-related injuries.

Injury Type	VR Protocol	Recovery Time	Patient Satisfaction	Compliance / Adherence
ACL reconstruction [96]	Virtual sports simulation & immersive feedback	Faster achievement of functional milestones (~1–2 weeks sooner than conventional rehab)	High; patients report greater engagement and motivation	High; gamified VR improves adherence

Rotator cuff repair [97]	3D movement training with VR feedback	Earlier functional gains in shoulder abduction; total rehab time not precisely quantified	High; VR improves patient motivation	Moderate to high; better adherence vs conventional therapy

Ankle sprain/ proprioception [98]	Balance challenge VR games	Faster improvement in balance and stability (measured via functional tests)	High; patients report enjoyment in interactive balance games	High; improved engagement vs standard balance exercises

Concussion / vestibular [99]	Cognitive-motor VR tasks	Accelerated improvement in vestibular/cognitive-motor function; specific % not reported	High; patients report satisfaction with interactive cognitive-motor tasks	High; patients complete sessions more consistently than conventional therapy

Abbreviations: VR: Virtual Reality, ACL: Anterior Cruciate Ligament

### Neural Network Applications in Neurorehabilitation

DT technology demonstrates strong effectiveness in neurological rehabilitation, where integration with VR environments enables intensive, task-specific training supported by deep learning algorithms [68, 100]. Controlled virtual environments facilitate high-frequency repetition of functional movements while providing precise feedback and continuous monitoring of progression through automated assessment pipelines [100, 101]. Within these frameworks, CNNs analyze patient movement patterns in real time, benchmark performance against normative reference databases, and identify specific motor deficits requiring targeted intervention [86, 102]. Reinforcement learning algorithms dynamically adapt task difficulty in response to patient performance, maintaining an optimal challenge point that promotes neuroplasticity while minimizing frustration and disengagement [102, 103].

Further advances are achieved through integration with brain–computer interfaces, which enable direct monitoring of neural signals during rehabilitation sessions and offer insights into cortical activation patterns and motor learning trajectories [103, 104]. Machine learning models applied to electroencephalographic and functional near-infrared spectroscopy data support individualized optimization of training protocols by aligning therapeutic demands with patientspecific neural response profiles [104, 105].

### Remote Rehabilitation Through IoT and Edge Computing

DT technology enables advanced remote rehabilitation programs that preserve therapeutic effectiveness while substantially improving accessibility by integrating IoT sensor networks and edge computing platforms [43, 106]. Home-based monitoring systems equipped with multimodal sensors continuously track patient progress and deliver real-time feedback, thereby reducing the need for constant in-person therapist supervision without compromising care quality [106, 107].

The underlying computational architecture relies on edge computing devices to perform initial data processing locally, minimizing latency and supporting real-time feedback loops that are critical for motor learning and functional rehabilitation [44, 108]. Within this framework, advanced anomaly detection algorithms automatically identify movement compensations, deviations from prescribed exercises, or adherence issues, triggering timely corrective interventions when needed [108, 109]. Integration with telehealth platforms further facilitates regular consultations with rehabilitation specialists via secure communication systems supporting high-definition video conferencing and real-time data exchange [109, 110].

These platforms are commonly linked to electronic health record systems, enabling seamless documentation, continuity of care, and longitudinal progress tracking across multidisciplinary healthcare teams [110, 111].

### Generative AI as a Natural Language Interface for DT Ecosystems

Beyond data processing and remote monitoring, the integration of Large Language Models (LLMs) represents a substantive shift toward more intuitive and interpretable athlete–system interaction within DT frameworks [105, 106]. In this context, Generative AI (GenAI) does not operate as a standalone advisory tool but rather functions as a natural language interface layered on top of the DT ecosystem. Within this architecture, the LLM is conditioned on athlete-specific physiological and metabolic data continuously updated within the DT environment [23, 106]. This configuration enables the translation of complex biomechanical and metabolic metrics into actionable, conversational feedback that is both context-aware and temporally relevant. Rather than generating generic training or nutritional recommendations, the GenAI layer interprets real-time signals from the so-called “metabolic twin” to deliver guidance that is syntactically fluent, physiologically grounded, and clinically aligned with the athlete’s individualized recovery and readiness state [23, 31].”

## ADVANCED AI AND GENERATIVE TECHNOLOGIES IN SPORTS ANALYTICS

### Generative AI and Large Language Models in Sports Medicine

The emergence of GenAI, exemplified by LLMs such as ChatGPT-4 and Claude, represents a transformative development in sports medicine, with implications for personalized training recommendations, clinical decision support, and athlete education [14, 112]. Within this context, GenAI should not be conceptualized as a standalone DT. Rather, it functions as an advanced interface layer embedded within the DT ecosystem, translating high-dimensional sensor-derived data into actionable natural-language insights that support practitioners’ decision-making. These systems demonstrate strong capabilities in processing natural language queries and generating contextually appropriate responses, thereby facilitating more accessible interactions among athletes, coaches, and clinical support staff [112, 113].

Recent evaluations of ChatGPT applications in resistance training prescription have highlighted both promising capabilities and critical limitations requiring systematic scrutiny [113, 114]. While algorithmic frameworks can generate training protocols grounded in established exercise science principles, concerns remain regarding accuracy, safety validation, and contextual appropriateness when applied to complex physiological scenarios, highlighting the need for continued empirical validation [114, 115].

The integration of GenAI with DT technology enables novel conversational coaching interfaces and real-time performance feedback systems [115, 116]. Natural language processing capabilities support intuitive, bidirectional communication between athletes and DT platforms, enhancing user engagement and facilitating system adoption [116, 117]. Advanced implementations further incorporate domain-specific knowledge bases that encompass exercise science literature, injury-prevention guidelines, and performance-optimization strategies, thereby improving contextual relevance and response fidelity [117, 118]. Nutritional counseling represents an additional emerging application area for GenAI in sports medicine, with growing empirical attention to the appropriateness and accuracy of AIgenerated dietary recommendations for athletic populations [113, 119]. Although these systems exhibit strong linguistic competence and general nutritional knowledge, persistent concerns regarding precision, safety, and clinical reliability necessitate rigorous validation before routine deployment [114, 120–122].

Further, a comprehensive assessment of ChatGPT-3.5 demonstrated its utility across multiple domains of medical writing and communication [123] The model was shown to enhance medical vocabulary and clarity, support accurate paraphrasing of complex content without informational loss, facilitate data interpretation, and enable efficient summarization of extensive scientific literature. Additionally, it proved effective in simplifying specialized medical language, improving accessibility for both patients and non-specialist professionals. Nevertheless, documented limitations, including occasional inaccuracies and contextual misinterpretations, underscored the need for expert human oversight. The authors concluded that while ChatGPT-3.5 can serve as a powerful assistive tool in medical communication and education, it cannot replace qualified medical professionals.

Accordingly, within the DT ecosystem, GenAI should be understood as an assistive, interface-level component rather than a fully autonomous DT. Although it enhances the interpretability and usability of complex computational models, it lacks the continuous real-time physiological synchronization that defines true DT architectures. Its role, therefore, remains supportive, reinforcing the centrality of human-in-the-loop governance in both clinical and performance-related decision-making [123].

### Machine Learning Integration with IoT Sensor Networks

The convergence of machine learning algorithms with IoT sensor networks has enabled the development of sophisticated monitoring ecosystems capable of processing large volumes of multimodal data in real time [124, 125]. As previously mentioned, contemporary implementations integrate a wide range of sensor modalities, including IMUs, physiological monitoring devices, environmental sensors, and biomechanical assessment tools, to capture complementary dimensions of athlete performance and context [124, 126].

Advanced data fusion techniques combine information across these heterogeneous sources to generate comprehensive athlete profiles that exceed the explanatory power of individual sensors [126, 127]. Within these pipelines, machine learning methods perform feature extraction to identify informative patterns in high-dimensional sensor streams, while ensemble approaches aggregate predictions from multiple models to enhance robustness and overall predictive accuracy [127, 128]. The underlying computational architecture increasingly relies on edge computing platforms that perform initial data processing at or near the sensor level, thereby reducing bandwidth demands and enabling low-latency, real-time feedback [108, 129]. In parallel, federated learning strategies enable distributed model training across decentralized sensor networks while preserving data privacy and security, thereby facilitating scalable and ethically compliant deployment of DT-enabled monitoring systems [129, 130].

### Deep Learning for Big Data Sports Analytics

The application of deep learning methodologies to big data analytics in sports enables the discovery of complex, non-linear patterns and relationships that remain inaccessible to traditional analytical approaches [28, 131]. Contemporary implementations routinely process multi-terabyte datasets comprising video footage, sensor-derived measurements, performance metrics, and contextual variables to generate actionable insights for performance optimization and decision support [131, 132]. CNNs are particularly effective in processing spatial data, including video streams and image sequences. In modern stadium-based settings, the integration of markerless optical tracking systems enables high-fidelity, automated capture of athlete kinematics. By applying computer vision algorithms to standard broadcast footage, these systems generate real-time skeletal representations that support continuous synchronization of the athlete’s DT without reliance on wearable sensors [33, 133].

To enhance computational efficiency and generalizability, such architectures frequently employ transfer learning strategies, in which pre-trained models are fine-tuned to sport-specific movement dynamics [134]. This approach facilitates the extraction of spatiotemporal parameters directly from competition footage, ensuring that the DT remains closely aligned with real-world performance. RNNs, particularly LSTM architectures, demonstrate strong capacity for modeling temporal dependencies in sports performance data [52, 135]. These models analyze sequential patterns in training-load exposure, recovery indicators, and performance outcomes to identify optimal training strategies and to anticipate future performance trajectories [135, 136].

## CURRENT CHALLENGES AND IMPLEMENTATION BARRIERS

### Technical Infrastructure and Computational Requirements

The implementation of DTs in sports environments is associated with substantial technical challenges, including the lack of standardized data protocols, limited validation of sensor accuracy, system integration complexity, and demanding computational infrastructure requirements [76, 137]. The marked heterogeneity of sensor technologies and data formats within IoT ecosystems imposes significant interoperability constraints that fundamentally limit system effectiveness, scalability, and cross-platform deployment [137, 138]. Moreover, as previously mentioned, sports generate data at exceptionally high rates, with individual athletes producing gigabytes of information daily, thereby necessitating advanced computational infrastructures capable of continuous, real-time processing and analysis [23, 139].

Meeting real-time processing requirements increasingly relies on sophisticated computational architectures, such as edge computing platforms, distributed processing frameworks, and high-performance computing clusters. However, the deployment and maintenance of these infrastructures often exceed the technical capacity and financial resources of many sports organizations [139, 140]. In parallel, the complexity inherent in multimodal data fusion demands specialized expertise in signal processing, machine learning, and software engineering, which often constitutes an additional, underappreciated barrier to implementation and sustainability [140, 141].

Data privacy and cybersecurity considerations further compound these challenges, particularly given the sensitive nature of athlete health data, performance metrics, and competitive intelligence [18, 142]. Ensuring compliance with international data protection regulations, including the General Data Protection Regulation (GDPR) and the Health Insurance Portability and Accountability Act (HIPAA), while preserving system usability and analytical functionality, requires a delicate balance between accessibility and security [142, 143]. The risks associated with data breaches, unauthorized access, and competitive espionage raise substantial ethical and legal concerns, underscoring the necessity for robust security architectures, encryption protocols, and governance frameworks tailored to DT-enabled sports ecosystems [143, 144].

### Economic and Accessibility Constraints

The substantial financial investment required for DT implementation introduces significant accessibility barriers, thereby limiting widespread adoption across diverse organizational settings [17, 145]. Initial capital expenditures encompass sensor acquisition, development of computational infrastructure, software licensing, and system integration services, with comprehensive deployments frequently exceeding several hundred thousand dollars [145, 146].

In addition to upfront costs, ongoing operational expenses, including system maintenance, software updates, technical support, and the employment of specialized personnel, further intensify the financial burden associated with DT deployment [146, 147]. The necessity of continuous hardware upgrades to maintain compatibility with rapidly evolving technologies and to accommodate expanding data volumes constitutes a sustained long-term investment commitment [147, 148].

Beyond financial constraints, the training requirements for effective DT utilization constitute a major barrier to implementation. Successful adoption necessitates structured education programs for athletes, coaches, technical staff, and healthcare professionals [20, 149]. Moreover, interpreting DT-generated outputs requires expertise in statistics and machine learning, as well as domain-specific knowledge, capabilities that are often not readily available within existing organizational frameworks, thereby further constraining scalability and equitable access [149, 150].

### Clinical Validation and Regulatory Considerations

Limited availability of long-term validation studies constrains confidence in the effectiveness and generalizability of DT applications across diverse athletic populations [15, 72]. Beyond questions of clinical utility, however, regulatory and ethical considerations constitute a major barrier to implementation. Under the GDPR, biometric and physiological information constitutes special category data pursuant to Article 9, obliging organizations to conduct Data Protection Impact Assessments to mitigate risks related to data breaches, misuse, or competitive espionage, while also safeguarding athletes’ rights to meaningful explanation of AI-driven decisions [151, 152].

Concurrently, unresolved tensions persist regarding data ownership and portability, as clubs often control the analysis of data while athletes retain legitimate claims to their biological and performance data. Emerging governance instruments, including FIFA’s Data Protection Regulations, have begun to address these issues by strengthening data portability provisions that allow athletes to retain their digital performance histories when transferring between teams or organizations [142, 153].

At the supranational level, the European Union (EU) Artificial Intelligence Act classifies health-related AI systems as high-risk, thereby imposing stringent requirements for documentation, bias mitigation, and transparency. These provisions are particularly salient in sports contexts, where algorithmic models must demonstrate validity across sex/gender, age, and performance *strata* and adhere to explicit human-in-the-loop requirements mandating clinical over-sight of AI-generated rehabilitation or training recommendations [153, 154].

Ethical governance is further shaped by alignment with the World Anti-Doping Agency (WADA) International Standard for the Protection of Privacy and Personal Information, which seeks to ensure that continuous physiological monitoring remains a tool for health protection and performance optimization rather than devolving into invasive surveillance practices that compromise athlete autonomy and dignity.

The convergence of these regulatory and ethical frameworks necessitates a shift away from opaque, black-box analytics toward transparent and explainable AI paradigms. As sports organizations advance toward comprehensive DT integration, priorities must extend beyond maximizing predictive accuracy to encompass methodological accountability, interpretability, and trustworthiness.

In this context, establishing standardized ethical oversight structures, analogous to institutional review boards or clinical trial governance committees, will be essential for managing the dual-use nature of DT technologies and ensuring that their deployment serves athlete empowerment and well-being rather than organizational exploitation [152, 154].

### Organizational Readiness and Practical Implementation Guidance

Beyond technical feasibility, the successful implementation of DT and AI-driven systems in sports environments requires a structured assessment of organizational readiness. Key readiness dimensions emerging from the identified implementation barriers include data maturity (availability of longitudinal, high-quality, and sufficiently large datasets [17, 23, 139]), technical infrastructure (edge or cloud computing capabilities, system interoperability [108, 139, 140]), human expertise (access to sports scientists, clinicians, and data engineers [20, 149]), and governance capacity (compliance with data protection, ethical oversight, and regulatory requirements [142, 152, 154]). Organizations lacking standardized data collection protocols, adequate computational resources, or interdisciplinary expertise may face substantial challenges when transitioning from experimental prototypes to operational deployment, regardless of reported model accuracy. Similarly, insufficient governance structures addressing data privacy, accountability, and human-inthe-loop oversight can significantly delay or prevent real-world implementation [142, 152].

Accordingly, before large-scale adoption, decision-makers should systematically evaluate readiness across these dimensions to determine whether DT technologies can be integrated into routine training, rehabilitation, and performance monitoring workflows in a sustainable, ethical, and effective manner.

## FUTURE DIRECTIONS AND EMERGING TECHNOLOGIES

### Next-Generation Computational Paradigms

Future DT development should prioritize integration with emerging computational paradigms, including quantum computing, neuromorphic processors, and advanced edge computing architectures, to expand processing capacity and support real-time analysis of increasingly complex and high-dimensional datasets [131, 155]. In particular, the application of quantum algorithms to optimization problems in training periodization and tactical decision-making represents a promising research avenue, with the potential to deliver substantial gains in computational efficiency and solution quality [155, 156].

Advances in AI, especially in natural language processing, computer vision, and autonomous systems, are expected to further enhance automated analysis and feedback capabilities within DT frameworks [116, 157]. The development of multimodal AI architectures capable of jointly processing video, audio, sensor, and textual data streams will enable more integrated and nuanced representations of athletic performance, behavior, and contextual dynamics [157, 158]. Parallel progress in standardization initiatives addressing data formats, communication protocols, and interoperability frameworks will be essential to facilitate system integration and support meaningful cross-platform comparison of DT implementations [138, 159]. Moreover, the expansion of open-source frameworks and collaborative research infrastructures is likely to accelerate methodological innovation while simultaneously reducing implementation costs and barriers to adoption [159, 160].

### Advanced Sensor Technologies and Data Acquisition

Next-generation sensor technologies, including flexible electronics, bio-integrated devices, and noninvasive monitoring systems, are expected to substantially enhance data fidelity while simultaneously reducing user burden [124, 161]. In particular, the development of smart textiles embedding distributed sensor networks will enable continuous, comprehensive biomechanical monitoring without constraining natural movement patterns or compromising athletic performance [161, 162]. Ongoing miniaturization trends in sensor engineering will further support the seamless integration of monitoring capabilities into standard sports equipment, such as footwear, protective gear, and sport-specific implements [162, 163]. Concurrent advances in materials science are anticipated to yield sensors with improved durability, measurement accuracy, and resistance to environmental stressors, rendering them suitable for deployment in demanding and variable sports contexts [163, 164].

In parallel, the integration of fifth-generation and forthcoming sixth-generation communication technologies will facilitate ultralowlatency data transmission and support large-scale IoT deployments within sports facilities and training environments [129, 165]. Enhanced connectivity will enable real-time coordination across distributed sensor networks and underpin advanced edge and distributed computing architectures, thereby further strengthening the responsiveness and scalability of DT-enabled monitoring systems [165, 166].

### Personalized Medicine and Precision Sports Science

The convergence of genomics, proteomics, and advanced analytical methods is expected to enable truly personalized applications in sports medicine by accounting for individual genetic predispositions, metabolic profiles, and injury susceptibility [57, 167]. Integrating multi-omics data streams with DT platforms will yield deeper insights into interindividual variability in responses to training, nutritional strategies, and recovery interventions [167, 168].

Advances in noninvasive biomarker sensing technologies will further support continuous monitoring of physiological status, inflammatory responses, and adaptation dynamics across training and competition cycles [81, 169].

Predictive models that incorporate genetic, environmental, and behavioral determinants are poised to substantially enhance injury prevention frameworks and performance optimization strategies by enabling earlier risk stratification and targeted intervention [61, 170].

Within this paradigm, precision sports medicine approaches will facilitate the development of individualized training and recovery protocols that maximize performance while minimizing health risks through evidence-based personalization [58, 171]. The integration of AI into personalized sports medicine is expected to yield adaptive systems that continuously refine recommendations in response to individual outcomes and evolving physiological states, thereby advancing the practical implementation of precision sports medicine [171, 172].

### Maturity Levels of Digital Twin Applications in Sports

DT applications vary widely in technological sophistication, dataset availability, validation rigor, and integration into operational workflows [23, 33, 43, 68, 115]. Some applications, such as real-time performance optimization and biomechanical tracking, have reached a high level of maturity and are proven reliable in elite sports contexts [33, 71, 173]. Others, including injury risk assessment models [61, 62], VR-based neurorehabilitation [55, 91], remote rehabilitation platforms [43, 106], and GenAI-driven metabolic monitoring [23, 105], show promising results in controlled studies but require additional validation, larger and more diverse datasets, and evaluation in operational environments before widespread adoption [2, 74, 115].

Table 5 summarizes these applications by current maturity level: “Validated and ready for implementation,” “Promising but requiring further validation,” and “Early research stage.”

**TABLE 5:** Maturity levels of digital twin applications in sports.

Digital Twin Application	Sport Context	Technical Readiness	Validation Status	Key Limitations
3D-CNN + LSTM for performance analysis	Basketball	Ready for deployment	Validated on large professional datasets (NBA SportVU)	May degrade on small or unconstrained datasets [33, 71]

SE-ResNet-CNN for multi-sport classification	General sports	Promising	Tested on curated datasets, limited field deployment	Needs real-world validation [33, 173]

Multi-scale CNN for swimming technique	Swimming	Early research	Proof-of-concept studies only	Small samples, laboratory-controlled environments [33]

Hybrid LSTM-CNN for gait analysis	Rehabilitation	Promising	Validated on moderate datasets	Limited generalizability to diverse populations [68, 71]

VR + DT rehabilitation environments	ACL, rotator cuff, ankle sprain	Ready for deployment	High patient engagement and adherence in clinical studies [55, 91]	Requires VR infrastructure and trained staff

IoT + Edge DT for remote rehab monitoring	Multiple sports	Promising	Limited deployment, pilot programs	Dependent on sensor availability, connectivity [43]

GenAI / LLMs as interface layer	Various	Early research	Initial pilots only	Needs rigorous empirical validation, contextual safety [105, 115]

## ADVANCES IN MONITORING TECHNOLOGIES FOR ATHLETIC PERFORMANCE WITH A FOCUS ON FOOTBALL

### Computer Vision: From Tactical Tracking to Biomechanical Digital Twins

Recent advances in deep learning–driven computer vision have enabled the automated extraction of tactical and locomotor metrics from conventional video recordings, marking a transition from basic two-dimensional player tracking toward sophisticated three-dimensional kinematic modelling.

CNNs and transformer-based architectures now support robust player and ball detection, spatiotemporal tracking, and near–realtime tactical analysis across competitive settings [173, 174]. For example, the PlayerTV framework generates player-centred highlights directly from broadcast footage [173], while commercial platforms such as ReSpoVision apply similar methodologies to quantify how movement efficiency and spatial behaviour influence match performance outcomes [71, 175]. At finer analytical resolution, the integration of markerless pose estimation constitutes a major methodological breakthrough, enabling the reconstruction of three-dimensional skeletal key points directly from video data. This capability allows practitioners to monitor joint-specific loading patterns and infer “virtual” mechanical stress during high-intensity actions, effectively enabling the construction of biomechanical DTs without the constraints imposed by physical markers or wearable systems [176].

### Optical and Physiological Sensing Modalities

In parallel with advances in movement assessment, optical sensing technologies have emerged as promising non-invasive modalities for monitoring biological signals in sports contexts. Established techniques such as near-infrared spectroscopy and photoplethysmography enable the assessment of physiological parameters, including heart rate and muscle oxygenation, during physical activity [74, 75]. More recently, remote photoplethysmography has enabled contactless monitoring of heart rate variability by detecting subtle skin-colour fluctuations associated with changes in blood volume [177]. Owing to their unobtrusive nature and compatibility with camera-based acquisition, these optical approaches are particularly well suited to high-intensity competitive environments, such as professional football, where wearable instrumentation may be impractical or disruptive.

### Sweat-based analyses

An emerging line of research centres on sweat biosensing, in which wearable patches incorporating microfluidic sensors enable continuous, non-invasive detection of biochemical markers. These systems support real-time monitoring of key metabolites, such as glucose and lactate, and electrolytes, including sodium and potassium, which collectively serve as informative proxies for hydration status and metabolic strain [178, 179]. Some platforms further enable quantification of stress-related biomarkers, notably cortisol, thereby extending their utility for assessing physiological stress responses [180]. By complementing external load metrics, sweat biosensing technologies offer critical insights into athletes’ internal physiological states, thereby enhancing the interpretability and precision of DTbased monitoring frameworks.

### Edge Computing and Real-Time Infrastructure

To address the processing bottlenecks associated with high-dimensional data streams, the sports technology ecosystem is increasingly adopting Edge AI solutions. Leveraging mobile edge computing architectures, lightweight neural network models can be deployed directly on sideline or venue-based servers to perform real-time inference [181]. This approach enables the instantaneous transformation of raw sensor signals into actionable performance metrics during competition, thereby eliminating the latency inherent in cloud-dependent processing pipelines [182, 183]. Such distributed architectures are essential for managing the high-velocity, low-latency data flows generated by modern stadium-scale sensor networks and for supporting time-critical decision-making in elite sports environments.

### Multimodal Data Fusion and Predictive Frameworks

The convergence of these technologies gives rise to an integrated monitoring ecosystem in which machine learning methods uncover latent structure across heterogeneous, high-dimensional data sources. To efficiently manage multi-terabyte data volumes, adaptive multiproxy mechanisms are employed to rapidly isolate salient biomechanical patterns and reduce computational complexity [184]. These representations are subsequently embedded in temporal graph neural network architectures that explicitly model player interactions and dynamic load propagation, enabling more accurate injury risk prediction and contextual performance analysis [185].

Final system-level synchronization is achieved through multimodal fusion transformers that align internal physiological load signals with external kinetic and kinematic data streams. This integration is further strengthened by specialized action-recognition algorithms that preserve robustness under occlusion and varying sensing conditions [186]. Collectively, these computational frameworks transform raw sensor outputs into clinically and operationally actionable insights, directly addressing contemporary research priorities in injury prevention, performance optimization, and evidence-based decision support for human performance systems [187, 188].

## CONCLUSION

The integration of DT technology represents a fundamental shift in sports science, moving athlete management from predominantly reactive approaches toward proactive, anticipatory strategies. This review demonstrates that DTs hold substantial potential for real-time performance optimization, predictive injury prevention, and personalized rehabilitation.

In applied settings, DTs function as advanced decision-support systems, enabling coaches to dynamically adjust training loads based on real-time fatigue simulations and allowing physiotherapists to employ virtual loading models to optimize recovery trajectories with greater precision and individual specificity. Despite these advances, a critical gap persists between the high accuracy achieved by laboratory-based models and their reliability in real-world sporting environments. Moreover, DT implementation raises complex ethical considerations, particularly regarding data sovereignty, continuous surveillance, and the potential psychological impact on athletes [189].

As the field evolves, the integration of next-generation computational paradigms, including neuromorphic processors, will be necessary to overcome current limitations in real-time processing and scalability. Achieving widespread and responsible adoption will require establishing standardized data-sharing protocols and robust ethical governance frameworks that clearly define data ownership, consent, and athletes’ rights. Furthermore, important knowledge gaps remain regarding the generalizability across diverse athlete populations, the long-term validation of DT models under field conditions, and the effective integration with emerging technologies such as generative AI, IoT, and multimodal sensor networks. Addressing these gaps is the most critical research priority for advancing the field responsibly and ensuring the practical, safe, and equitable implementation. Ultimately, while the concept of the virtual athlete has transitioned from theory to practice, its transformative impact will depend on maintaining a careful balance between computational sophistication, methodological transparency, and ethical integrity.
